# Integrated Machine Learning Algorithms-Enhanced Predication for Cervical Cancer from Mass Spectrometry-Based Proteomics Data

**DOI:** 10.3390/bioengineering12030269

**Published:** 2025-03-09

**Authors:** Da Zhang, Lihong Zhao, Bo Guo, Aihong Guo, Jiangbo Ding, Dongdong Tong, Bingju Wang, Zhangjian Zhou

**Affiliations:** 1 Department of Oncology, The Second Affiliated Hospital, Xi’an Jiaotong University, Xi’an 710000, China; zhangda581012@163.com; 2 Department of Dermatology, The Second Affiliated Hospital, Xi’an Jiaotong University, Xi’an 710000, China; zhaolh1993@163.com; 3 Department of Cell Biology and Genetics, School of Basic Medical Sciences, Xi’an Jiaotong University Health Science Center, Xi’an 710000, China; bo_guo@xjtu.edu.cn (B.G.); guoaihong2021@163.com (A.G.); dingjiangbo2022@163.com (J.D.); tongdd@xjtu.edu.cn (D.T.); 4 Department of Clinical Research, Xianyang Hospital of Yan’an University, Xianyang 712000, China; 5 Department of Clinical Research, Rugao Hospital of Shenzhen Jingcheng Medical Group, Rugao 226500, China

**Keywords:** artificial intelligence, proteomics, cervical cancer, early diagnosis

## Abstract

Early diagnosis is critical for improving outcomes in cancer patients; however, the application of diagnostic markers derived from serum proteomic screening remains challenging. Artificial intelligence (AI), encompassing deep learning and machine learning (ML), has gained increasing prominence across various scientific disciplines. In this study, we utilized cervical cancer (CC) as a model to develop an AI-driven pipeline for the identification and validation of serum biomarkers for early cancer diagnosis, leveraging mass spectrometry-based proteomics data. By processing and normalizing serum polypeptide differential peaks from 240 patients, we employed eight distinct ML algorithms to classify and analyze these differential polypeptide peaks, subsequently constructing receiver operating characteristic (ROC) curves and confusion matrices. Key performance metrics, including accuracy, precision, recall, and F1 score, were systematically evaluated. Furthermore, by integrating feature importance values, Shapley values, and local interpretable model-agnostic explanation (LIME) values, we demonstrated that the diagnostic area under the curve (AUC) achieved by our multi-dimensional learning models approached 1, significantly outperforming the diagnostic AUC of single markers derived from the PRIDE database. These findings underscore the potential of proteomics-driven integrated machine learning as a robust strategy to enhance early cancer diagnosis, offering a promising avenue for clinical translation.

## 1. Introduction

Cervical cancer represents the fourth most prevalent malignancy among women worldwide, with an estimated 660,000 new cases and 350,000 deaths reported in 2022 [1]. In China, cervical cancer accounted for 150,700 new cases in 2022, ranking as the fifth most common cancer among females, while the mortality rate reached 55,700 cases, positioning it as the sixth leading cause of cancer-related deaths in women [2]. These epidemiological data underscore the persistently high burden of cervical cancer, particularly in developing countries where access to effective screening programs remains limited [3]. Early diagnosis plays a critical role in the management of cervical cancer, as it significantly enhances the cure rates and improves patient outcomes [4]. Current clinical diagnostic approaches for cervical cancer include cervical cytology [5], human papillomavirus (HPV) testing [6], colposcopy [7], cervical biopsy [8], and imaging techniques [9]. While these methods, either individually or in combination, demonstrate relatively high accuracy in detecting cervical cancer and precancerous lesions, they are associated with notable limitations. These include suboptimal sensitivity, stringent technical requirements for practitioners, and the potential for secondary injury due to invasive procedures [10,11]. Consequently, the development of novel diagnostic technologies holds significant promise for further enhancing the accuracy and efficiency of cervical cancer detection.

The application of proteomics to the discovery of novel tumor biomarkers represents a pivotal strategy in cancer screening. This approach involves identifying specific biomarkers in body fluids, such as blood and urine, to detect the presence of tumors [12,13]. While several tumor biomarkers, including carcinoembryonic antigen (CEA) for colorectal cancer screening [14], carbohydrate antigen 125 (CA125) for ovarian cancer [15], and prostate-specific antigen (PSA) for prostate cancer [16], have shown utility in longitudinal disease monitoring, their clinical application remains limited. Notably, the number of tumor biomarkers approved by the U.S. Food and Drug Administration (FDA) is restricted, and proteomics-based biomarker detection continues to face significant challenges. The specificity and sensitivity of individual tumor biomarkers are often suboptimal, as elevated levels can also occur in non-cancerous conditions, leading to false positive results. Although the combined use of multiple biomarkers can enhance diagnostic accuracy to some extent, it still fails to achieve the desired efficacy for early screening, with instances of missed diagnoses persisting [17,18,19]. Furthermore, the development of antibody-dependent diagnostic kits based on tumor biomarkers encounters additional obstacles such as difficulties in producing high-quality antibodies, poor stability, and high costs associated with development and commercialization [20,21].

The rapid advancement of artificial intelligence (AI) has revolutionized cancer screening by offering innovative approaches to disease detection. AI technologies, particularly machine learning (ML), possess the capability to analyze multi-dimensional datasets derived from genomics, transcriptomics, metabolomics, and imaging, thereby facilitating the identification of complex patterns and biomarkers associated with tumors [22,23,24]. For instance, deep learning algorithms have been successfully applied to analyze pathological images and radiological data, significantly enhancing the diagnostic accuracy of breast cancer [25,26]. Similarly, in prostate cancer diagnosis, AI systems have demonstrated the ability to precisely identify malignant regions by analyzing magnetic resonance imaging (MRI) data, even in cases where cancerous lesions are not readily discernible, thereby providing critical diagnostic insights [27,28]. Building on these advancements, the present study developed an AI-driven pipeline integrating machine learning with mass spectrometry-based proteomics to identify diagnostic markers, using cervical cancer as a model. By analyzing the serum data from 240 patients with eight ML algorithms, our multi-dimensional models achieved near-perfect diagnostic accuracy (AUC ≈ 1), significantly outperforming single-marker approaches and demonstrating robust clinical potential. This research aims to further advance the development and application of antibody-independent detection methods, offering a promising alternative for early and accurate cancer diagnosis.

The remaining sections of the paper are summarized as follows. In Section 2, we detail the prospective case–control cohort design, standardized serum collection protocols, and stringent exclusion criteria for cervical cancer patients and healthy controls. We systematically delineate the mass spectrometry-based proteomics workflow, encompassing raw data preprocessing, feature extraction, dimensionality reduction, and the implementation of eight ML classifiers. Feature selection strategies integrating model-specific importance scores, Shapley values, and LIME interpretations are rigorously described, alongside unsupervised clustering and decision curve analysis for clinical utility assessment. Section 3 presents the identification of differentially expressed serum peptides via MALDI-TOF MS, with ML models achieving near-perfect diagnostic accuracy. Key biomarkers were functionally validated through LC-ESI-MS/MS and bioinformatics analyses, revealing associations with immune response, complement activation, and coagulation cascades. Section 4 critically evaluates the clinical translatability of the AI-proteomics pipeline, addresses limitations in dataset heterogeneity, and proposes future directions for multi-omics integration and multi-center validation. Section 5 contextualizes the study within existing ML-driven cancer diagnostics, emphasizing the superiority of multi-dimensional biomarker panels over single-marker approaches and underscoring the paradigm-shifting potential of proteomics-enhanced AI for early cervical cancer detection.

## 2. Materials and Methods

### 2.1. Serum Proteomics Workflow Summary

This analysis involved MALDI-TOF-based serum proteomic profiling. Raw spectral data underwent curve-fitting to resolve characteristic protein/peptide peaks, followed by normalization to reduce technical variability. A normalized peak intensity matrix was analyzed with machine learning classifiers to identify discriminative features. Peaks with high feature importance were prioritized for biological annotation to identify validated biomarkers. All data analysis and visualization were performed using Python 3.7, with plotting conducted using Matplotlib (version 3.7.0) and Seaborn (version 0.13.2).

#### 2.1.1. MALDI-TOF-Based Proteomic Profiling and Mass Spectrometry Data Preprocessing for Serum Samples

##### Patients and Samples

A prospective case–control study was conducted at Xianyang Hospital, Yan’an University involving 240 cervical cancer patients and 249 age- and sex-matched healthy controls. Patients were recruited between January 2023 and September 2024 following histopathological confirmation of cervical cancer. Exclusion criteria included prior chemotherapy, radiotherapy, or comorbidities affecting the serum protein profiles (e.g., chronic kidney disease). Healthy donors were screened via questionnaires and routine blood tests to exclude underlying conditions. Blood samples (5 mL) were collected in vacuum blood collection tubes without anticoagulants, allowed to clot at 4 °C for 1 h, and centrifuged (3000× *g*, 15 min, 4 °C). Serum aliquots (500 μL) were stored at −80 °C within 2 h post-collection to minimize protein degradation. The study protocol (YDXY-KY-2023-004) adhered to the Declaration of Helsinki, with written informed consent obtained from all participants.

##### Serum Sample Processing and MALDI-TOF MS Analysis

Serum peptides were enriched using the MB-IMAC Kit (Yuan Tong Hui Ze Biotechnology, Xi’an, Shaanxi Province, China) to isolate low-abundance biomarkers. Magnetic beads (20 µL) were pre-equilibrated with binding buffer (20 µL) and mixed with serum (20 µL) for 5 min at room temperature. Three sequential washes with 100 µL wash buffer removed nonspecific binders, followed by elution in 10 µL elution buffer stabilized with 10 µL stabilization buffer. For MALDI-TOF MS analysis, 1 µL of eluate was co-crystallized with the α-cyano-4-hydroxycinnamic acid matrix (3 mg/mL in 50% acetonitrile/2% trifluoroacetic acid) on a stainless-steel target. Triplicate spots per sample were analyzed using a Zybio EXS2600 MALDI-TOF mass spectrometer (Chongqing, China) in linear positive ion mode (laser frequency: 1000 Hz; mass range: 1000–15,000 Da). External calibration was performed weekly using a peptide/protein standard mixture (Bruker Daltonics, Bremen, Germany). To minimize bias, sample processing and MS acquisition were randomized across groups, with operators blinded to clinical status.

##### Raw Data Preprocessing

Raw spectral data were preprocessed using a Python pipeline integrating numpy (1.21.5) [29], scipy (1.7.3) [30], and scikit-learn (1.0.2) [31]. Intensity values were extracted from m/z spectra and smoothed using a Savitzky–Golay filter (window size = 21, polynomial order = 10) to preserve the peak morphology while reducing high-frequency noise. A moving median filter (window = 10) further suppressed sporadic noise spikes. Baseline correction was performed via morphological opening (white_tophat function, structuring element size = 11) to isolate true signals from the background drift. Processed spectra were visually validated using Matplotlib (version 3.7.0) to ensure alignment between the detected peaks and raw spectral features.

##### Peak Feature Extraction, Normalization, and Integrative Data Matrix Construction

Significant *m*/*z* peaks were identified using scipy.signal.find_peaks with adaptive thresholds: minimum height = 3% of maximum intensity, and minimum peak width = 5 data points. Detected peaks were validated by overlaying peak markers on raw spectra excluding artifactual peaks near matrix cluster regions (*m*/*z* 1500–2000). Cross-sample alignment used an iterative greedy algorithm with a mass tolerance of 2500 ppm, accounting for instrumental drift (±1500 ppm) and biological variation. The final feature matrix (samples × peaks) encoded relative peak areas, with missing values imputed as zeros for undetected peaks or linearly interpolated from neighboring m/z bins.

#### 2.1.2. Machine Learning-Driven Proteomic Data Analysis: From Dimensionality Reduction to Biomarker Candidate Prioritization

##### Dimension Reduction Analysis

Dimensionality reduction analysis was conducted on the dataset using principal component analysis (PCA), kernel principal component analysis (KPCA), and t-distributed stochastic neighbor embedding (t-SNE). The pandas library (1.3.5) [32] was employed to read the data, while numpy facilitated the numerical computations. The dimensionality reduction algorithms were implemented using the relevant modules in the sklearn library. For visualization, matplotlib.pyplot (3.5.2) [33] and seaborn’s kdeplot (0.11.2) [34] were utilized to display the data distribution density, enabling the exploration of data values from multiple perspectives for comprehensive analysis.

##### Machine Learning

The dataset was partitioned into training (80%) and test (20%) sets via stratified sampling, preserving class distributions. Eight classifiers were evaluated:

Traditional: KNN, SVM, Gaussian Naive Bayes, decision tree.

Ensemble: Random forest, XGBoost, AdaBoost, LGBM (3.3.2) [35].

Model performance was assessed via 5-fold stratified cross-validation, with metrics including AUC-ROC, accuracy, precision, recall, and F1 score.

##### Feature Selection

Three complementary approaches identified the biologically relevant peptides:

Model-based importance: Random forest and XGBoost (1.6.1) [36] provided the Gini importance scores.

SHAP values (0.41.0) [37]: KernelExplainer computed the marginal feature contributions using 200 background samples.

LIME (0.2.0.1) [38]: LimeTabularExplainer generated instance-specific explanations, aggregated into global rankings.

The top 20 consensus features were prioritized for downstream validation, with discordant results resolved by majority voting.

### 2.2. Annotated Biomarker-Driven Machine Learning and Clinical Validation for Cervical Cancer Diagnostics

Key mass spectral peaks contributing to classifier performance were biologically annotated via MS/MS (peptides/proteins). A relative abundance matrix was reconstructed and analyzed through unsupervised clustering (bisecting K-means, BIRCH) and UMAP visualization to compare the clustering patterns between the annotated matrix and original data. Subsequently, clinical validation analyses were conducted on individual biomarkers, encompassing ROC optimization (Youden index-driven thresholding) and decision curve analysis (DCA) to quantify the diagnostic accuracy and clinical net benefit. Finally, machine learning classifiers (as described in previous sections) were utilized to evaluate the classification efficacy of the biologically annotated matrix in distinguishing cervical cancer from healthy samples.

#### 2.2.1. Integrated Proteomic Data Processing: High-Resolution Peptide Identification via Orbitrap MS and Unsupervised Clustering for Pattern Discovery

##### Peptide Identification

Eluted peptides were separated on a nanoACQUITY UPLC (Waters, Milford, MA, USA) with a 60-min gradient (5–80% acetonitrile) and analyzed on an Orbitrap Fusion Lumos (Thermo Fisher, Waltham, MA, USA). MS1 scans (*m*/*z* 200–2000) were acquired at 100,000 resolution, with the top 10 precursors selected for HCD fragmentation (25% CE). Data were searched against UniProt (https://www.uniprot.org/) (accessed on 4 December 2024) [39] using Proteome Discoverer 2.5 (precursor tolerance = 20 ppm; fragment tolerance = 1.0 Da). The false discovery rate (FDR) was controlled at <1% using the target-decoy strategy.

##### Unsupervised Clustering

Bisecting K-means partitioned the data via recursive binary splitting, minimizing the within-cluster sum of squared distances. BIRCH constructed a clustering feature (CF) tree (threshold = 0.5, branching factor = 50) to handle large datasets efficiently. Cluster quality was quantified using the Rand index and adjusted mutual information (AMI) against clinical labels. Results were visualized via UMAP projections.

#### 2.2.2. Comprehensive Biomarker Evaluation: ROC-Driven Threshold Optimization and Decision Curve Analysis for Clinical Utility Validation

##### Roc Evaluation of Unique Indicator

The pandas and numpy libraries were used for data manipulation and numerical computations. ROC analysis was performed using sklearn.metrics modules (roc_curve, auc). For standard evaluation, the FPR, TPR, and thresholds were computed via the roc_curve, with AUC derived from the auc function. ROC curves visualized the discriminative performance between groups.

For threshold optimization, 100 equally spaced thresholds were generated (*np.linspace*). At each threshold, the sensitivity (SEN), specificity (SPEC), and Youden index were calculated via confusion matrices. The threshold maximizing YI was identified as the optimal discrimination point, marked on ROC plots to highlight peak performance.

##### Decision Curve Analysis

A 5-fold stratified cross-validation framework was implemented for model evaluation. In each iteration, the training set was partitioned into training and validation subsets. Classifiers were trained on the training subset, with prediction probabilities generated for the validation subset.

Net benefit calculation:

Model net benefit:

$$Net\;Benefit=\frac{True\;Positives}{Total\;Samples}-\frac{False\;Positives}{Total\;Samples}\times \left(\frac{threshold}{1-threshold}\right)$$

Treat-all strategy:

$$\begin{array}{cc} Net\text{\_}benefit\text{\_}all & \\ & =\frac{True\;Positives}{True\;Positives+True\;Negatives}\\ & -\frac{False\;Positives}{True\;Positives+True\;Negatives}\times \left(\frac{threshold}{1-threshold}\right) \end{array}$$

Visualization and analysis: Net benefit curves with 95% confidence intervals were plotted using the mean and standard deviation across folds. Simpson’s method quantified the area where the model outperformed “treat-none” (net benefit > 0).

Single-feature thresholding: Indicators were classified as highly expressed (cancer mean > healthy mean) or lowly expressed. Thresholds were set above (highly expressed) or below (lowly expressed) the indicator’s value range to optimize net benefit.

### 2.3. Bioinformatics Analysis and Clinical Validation of Public Data

Biologically annotated biomarkers underwent KEGG/GO enrichment to uncover cervical cancer-associated pathways. ROC and decision curve analysis (DCA) using public proteomic data further assessed their diagnostic accuracy and clinical utility.

#### 2.3.1. Bioinformatics Analysis

Gene Ontology (GO) and KEGG pathway enrichment analyses were performed using ClusterProfiler (4.12.6) [40].

#### 2.3.2. Clinical Validation of Public Data

Candidate biomarkers were validated against the PRIDE database (https://www.ebi.ac.uk/pride/) (accessed on 5 January 2025) [41] under the project accession number PXD055203. Data-independent acquisition (DIA) mass spectrometry analysis was utilized to evaluate the receiver operating characteristic (ROC) curves and differential abundance criteria (DAC) values of target proteins.

## 3. Results

### 3.1. General Information

The study protocol was approved by the Ethics Committee of Xianyang Hospital, Yan’an University (Approval No.: YDXY-KY-2023-004). The study cohort consisted of 240 cervical cancer patients with a median (IQR) age of 49 (37, 59) years, and 249 healthy donors with a median (IQR) age of 48 (33, 65) years. The Mann–Whitney U test, a non-parametric test, was employed to compare the ages of the two groups. The results indicated no significant difference in age distribution between the two groups (U = 2.991 × 10^4^, *p* = 0.9865). In this study, the significance level was set at α = 0.05. When *p* > 0.05, the difference was considered not statistically significant.

### 3.2. Mass Spectrometry Data Processing and Differential Polypeptide Screening

**Figure 1:**
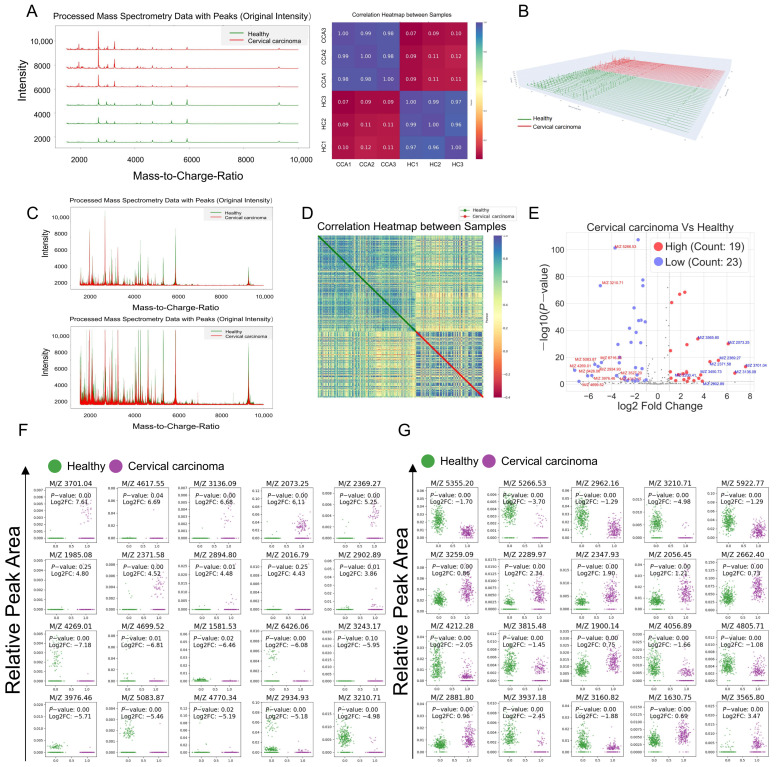
Mass spectrometry data processing and the screening of differential peptides. (**A**) LC-MS identification of peptide profiles and Pearson coefficients within the 1000–10,000 KDa range. (**B**) 3D fitting image of differential peptide peaks. (**C**) Top: Overlaid mass spectrum peaks after fitting of raw time-of-flight mass spectrometry data; Bottom: Normalized overlaid mass spectrum peaks of all samples. (**D**) Correlation analysis between samples, green: healthy samples, red: cervical cancer samples. (**E**) Volcano plot of differential serum peptides between the cervical cancer patients and healthy donors, red: upregulated, blue: downregulated. (**F**) Top 20 peptides with the largest expression differences (log_2_FC). (**G**) Top 20 peptides with the largest statistical differences (*p* value).

Initially, serum polypeptide extraction kits were employed to isolate polypeptides from the peripheral blood of cervical cancer patients and healthy donors. Polypeptide profiles within the range of 1000–10,000 KDa were identified using MALDI-TOF MS, and triplicate measurements of the same sample demonstrated excellent reproducibility (Figure 1A). Subsequently, 3D fitting images of the differential polypeptide peaks revealed that the majority of peak values distinguishing the two groups were distributed within the range of 1500–6000 KDa (Figure 1B). The mass spectrometry peaks from the original time-of-flight mass spectrometry data were overlaid after fitting and normalization, respectively (Figure 1C). Correlation analysis between samples indicated that inter-group correlations were lower than the intra-group correlations (Figure 1D). A volcano plot was generated to visualize the statistical results of differential serum polypeptides between the cervical cancer patients and healthy donors. The analysis identified 19 upregulated and 23 downregulated polypeptide peaks in the peripheral blood serum of cervical cancer patients compared with healthy donors (Figure 1E, *p* < 0.001, |log_2_FC| > 1). Based on these findings, the top 20 peptides with the largest expression differences (Figure 1F) and the top 20 peptides with the most significant statistical differences (Figure 1G) were selected for further investigation to explore their potential diagnostic value.

### 3.3. Evaluation of Diagnostic Efficiency Based on Different Machine Learning Algorithms

**Figure 2:**
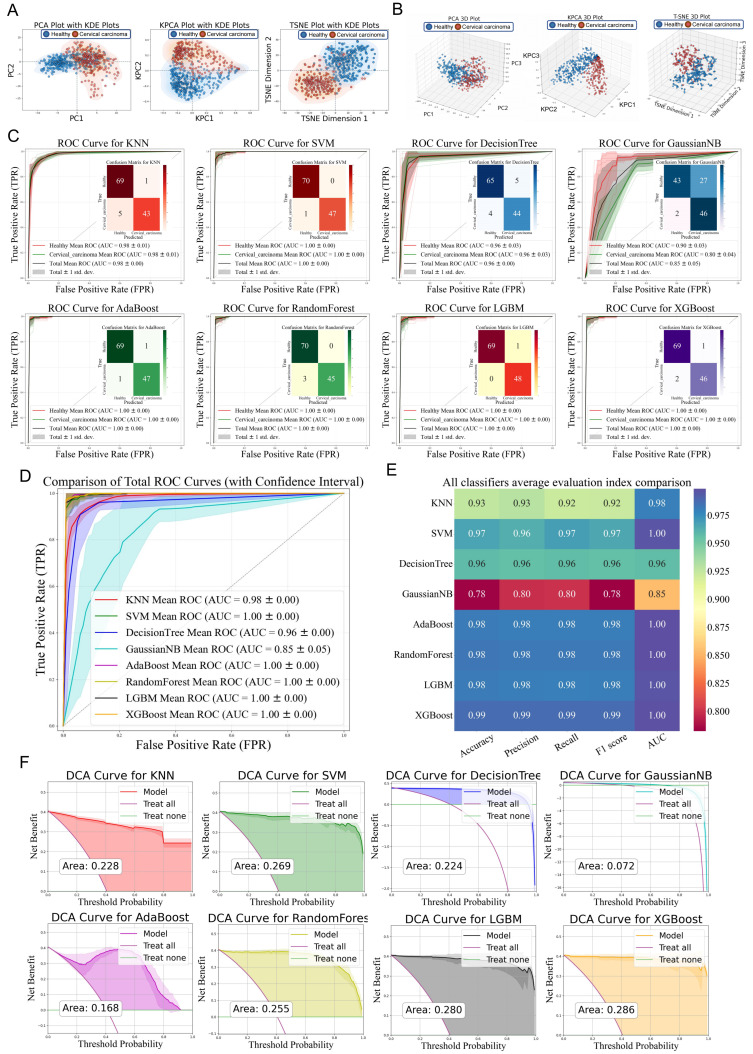
Evaluation of diagnostic efficacy using different machine learning algorithm. (**A**,**B**) 2D/3D visualization of mass spectrometry data after dimensionality reduction using PCA, KPCA, and t-SNE. The shaded regions represent the distribution areas of samples, demonstrating their spatial dispersion. (**C**) ROC curves and confusion matrices for the classification of differential peptide peaks between the two groups using eight different machine learning algorithms. The shaded regions represent the variability (confidence intervals) of performance metrics across cross-validation iterations, illustrating model robustness. The confusion matrix quantifies classification outcomes: TP (top-left) and TN (bottom-right) on the main diagonal indicate correct predictions, while FP and FN on the off-diagonal denote misclassifications. (**D**) Integration of different ROC curves and their association with TPR and FPR. (**E**) Performance metrics including accuracy, precision, recall, and F1 score for different machine learning algorithms. (**F**) DCA curves for different machine learning algorithms. The shaded regions highlight the net benefit confidence intervals across clinical threshold probabilities, demonstrating superior clinical utility compared with the treat-all and treat-none strategies within the optimal threshold range.

We conducted dimensionality reduction analysis on the aforementioned mass spectrometry data using principal component analysis (PCA), kernel principal component analysis (KPCA), and t-distributed stochastic neighbor embedding (t-SNE). The resulting data distribution density was visualized using matplotlib.pyplot and seaborn’s kdeplot to generate 2D/3D plots, enabling an exploration of the data from multiple perspectives for comprehensive analysis and decision-making (Figure 2A,B). Following the division of the dataset into training and test sets, eight distinct machine learning models were employed to classify the two groups of differential polypeptide peaks. The performance of each model was evaluated using ROC curves and confusion matrices (Figure 2C). The mean AUC values under different algorithms were as follows: Total mean AUC = 0.98 ± 0.00 (Healthy AUC = 0.98 ± 0.01, Cervical AUC = 0.98 ± 0.01), SVM: Total mean AUC = 1.00 ± 0.00 (Healthy AUC = 1.00 ± 0.00, Cervical AUC = 1.00 ± 0.00), Decision tree: Total mean AUC = 0.96 ± 0.00 (Healthy AUC = 0.96 ± 0.03, Cervical AUC = 0.96 ± 0.03), Gaussian NB: Total mean AUC = 0.85 ± 0.05 (Healthy AUC = 0.90 ± 0.03, Cervical AUC = 0.80 ± 0.04), AdaBoost: Total mean AUC = 1.00 ± 0.00 (Healthy AUC = 1.00 ± 0.00, Cervical AUC = 1.00 ± 0.00), Random forest: Total mean AUC = 1.00 ± 0.00 (Healthy AUC = 1.00 ± 0.00, Cervical AUC = 1.00 ± 0.00), LGBM: Total mean AUC = 1.00 ± 0.00 (Healthy AUC = 1.00 ± 0.00, Cervical AUC = 1.00 ± 0.00), and XGBoost: Total mean AUC = 1.00 ± 0.00 (Healthy AUC = 1.00 ± 0.00, Cervical AUC = 1.00 ± 0.00). These ROC curves were integrated to illustrate their correlations with the true positive rate (TPR) and false positive rate (FPR), reflecting the discriminative ability of each model (Figure 2D). Misclassification arose from the substantial biomarker profile overlap between the healthy controls and cervical cancer cohorts, attributable to complex protein expression dynamics and conserved biological pathways. Key contributors include: (1) biological heterogeneity and technical variability obscuring inter-group distinctions, (2) feature redundancy due to peptides with comparable abundance patterns across cohorts, and (3) algorithmic constraints in resolving subtle discriminative signatures within high-dimensional proteomic data, ultimately diminishing model specificity. Key performance metrics, including accuracy, precision, recall, and F1 score, were calculated to quantitatively evaluate the model performance across multiple dimensions, facilitating the selection of the optimal model for further research (Figure 2E). Finally, decision curve analysis (DCA) was performed to quantify the net benefits of the prediction models under varying decision thresholds. The DCA curves demonstrated that the net benefits of all models exceeded the reference lines (including “treat-none” and “treat-all” strategies). Notably, the machine learning classifiers exhibited a lower yield rate in the clinical diagnosis of cervical cancer, highlighting their potential for improved diagnostic efficiency (Figure 2F).

### 3.4. Feature Selection of Different Machine Learning Algorithms

**Figure 3:**
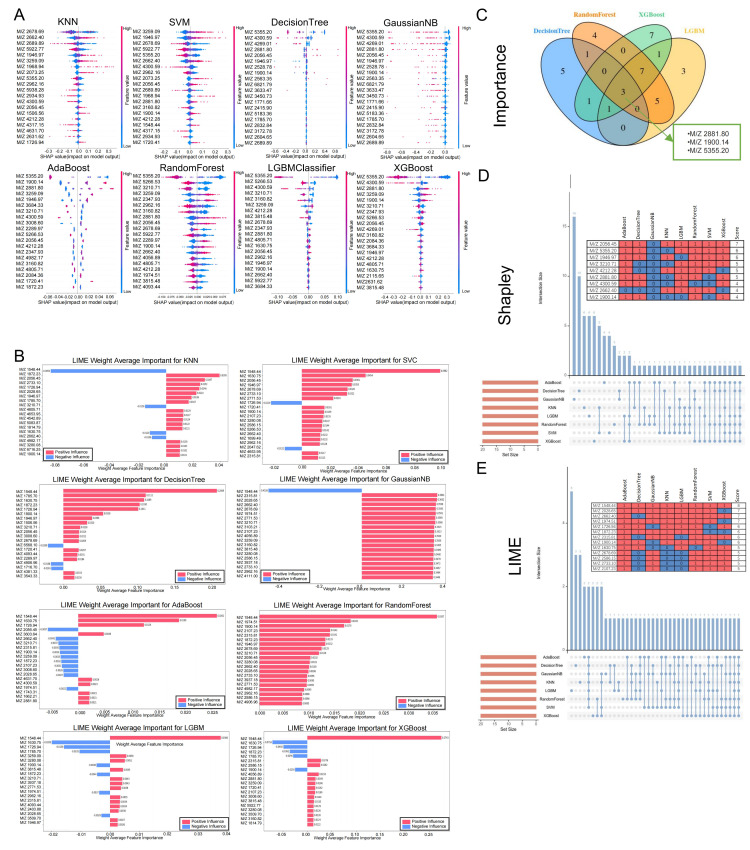
Feature selection in different machine learning models. (**A**,**B**) High-performing differential peptides identified based on feature value extraction using different machine learning algorithms. (**C**) Feature importance values extracted and cross-validated by the decision tree, random forest, LGBM, and XGBoost classifiers. (**D**) Shapley and (**E**) LIME analysis for different machine learning algorithms.

In the machine learning workflow, the screening of multiple importance indicators provides deeper insights into model behavior. To this end, we initially extracted feature importance values from eight distinct models and identified differential polypeptides that exhibited strong performance across different computational models (Figure 3A,B). Subsequently, classifiers with the feature_importances_ attribute, such as decision tree, random forest, light gradient boosting machine (LGBM), and extreme gradient boosting (XGBoost), were prioritized. Feature importance values were directly extracted and cross-validated, yielding three significant differential polypeptide peaks: M/Z = 2881.80, M/Z = 1900.14, and M/Z = 5355.20 (Figure 3C).

Shapley values were employed to precisely quantify the marginal contribution of each feature to the prediction of individual samples. A random subset of 200 samples from the training set was selected as background data. The shap.KernelExplainer function, combined with the model function and background data, was used to construct an explainer, and Shapley values for the training set under different models were calculated (Figure 3D). Additionally, LIME values were computed and sorted across different models. Feature indices with the highest absolute importance values were identified, and their corresponding feature names and importance values were extracted. The analysis results of LIME values, including their positive and negative influences, were presented (Figure 3E). By integrating the three importance indicators—feature importance values, Shapley values, and LIME values—we gained comprehensive insights into the model behavior from multiple dimensions, facilitating the optimization of decision-making strategies.

### 3.5. Analysis of Clinical Indicators of Single Differential Polypeptide

**Figure 4:**
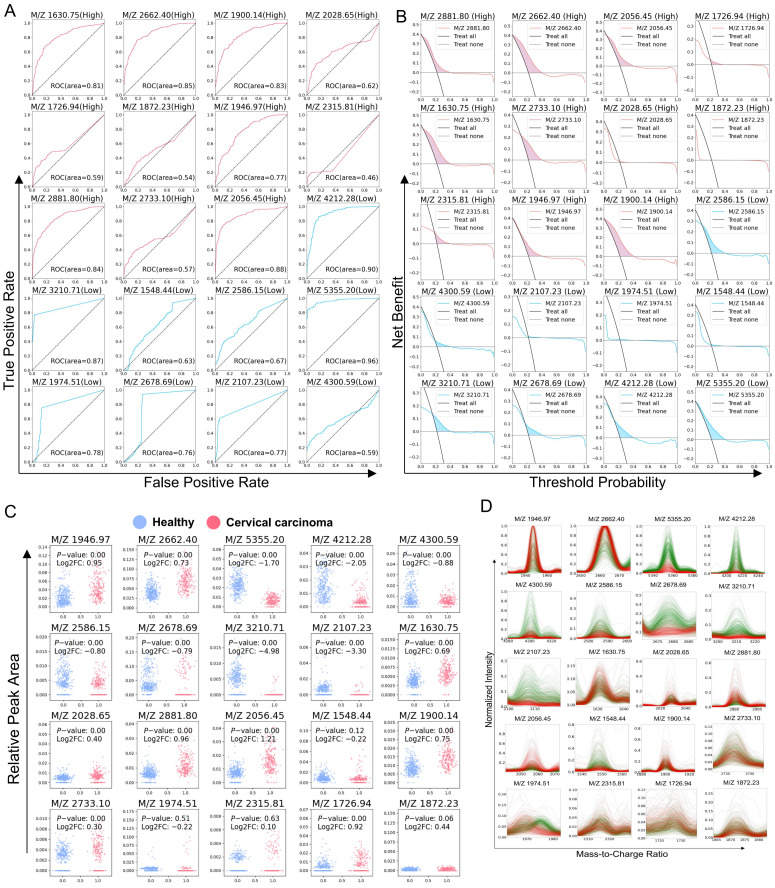
Analysis of clinical indicators of a single differential polypeptide. (**A**) ROC curve of a single differential polypeptide, red: upregulated peptide, blue: downregulated peptide. (**B**) DCA curve of a single differential polypeptide and its evaluation against the reference line, red: upregulated peptide, blue: downregulated peptide. (**C**) Expression analysis of a single differential polypeptide. (**D**) Normalized expression value spectrum peak overlay of a single differential polypeptide.

To further evaluate the clinical significance of the identified features that demonstrated significant discriminatory power across different models in cervical cancer, we first generated ROC curves for each feature individually. The results revealed varying areas under the curve (AUC) for different feature indicators (Figure 4A) including M/Z = 2056.45 (AUC = 0.88), M/Z = 1630.75 (AUC = 0.81), M/Z = 2881.80 (AUC = 0.84), M/Z = 2662.40 (AUC = 0.85), M/Z = 3210.71 (AUC = 0.87), M/Z = 5355.20 (AUC = 0.96), and M/Z = 4212.28 (AUC = 0.90). Decision curve analysis (DCA) was subsequently performed for each feature, and the corresponding curves were plotted (Figure 4B). By comparing the model prediction curves of each feature with the reference lines (including “treat-none” and “treat-all”) as shaded areas, the net benefit values of each feature as diagnostic indicators were determined. The analysis revealed statistically significant differences in the expression levels of the selected peptides such as M/Z = 2056.45, M/Z = 5355.20, M/Z = 3210.71, M/Z = 1900.14, and M/Z = 2881.80 (Figure 4C). Additionally, the normalized mass spectrometry peak plots of these features were presented (Figure 4D).

### 3.6. Identification of Differential Polypeptides and Evaluation of Their Diagnostic Efficacy

**Figure 5:**
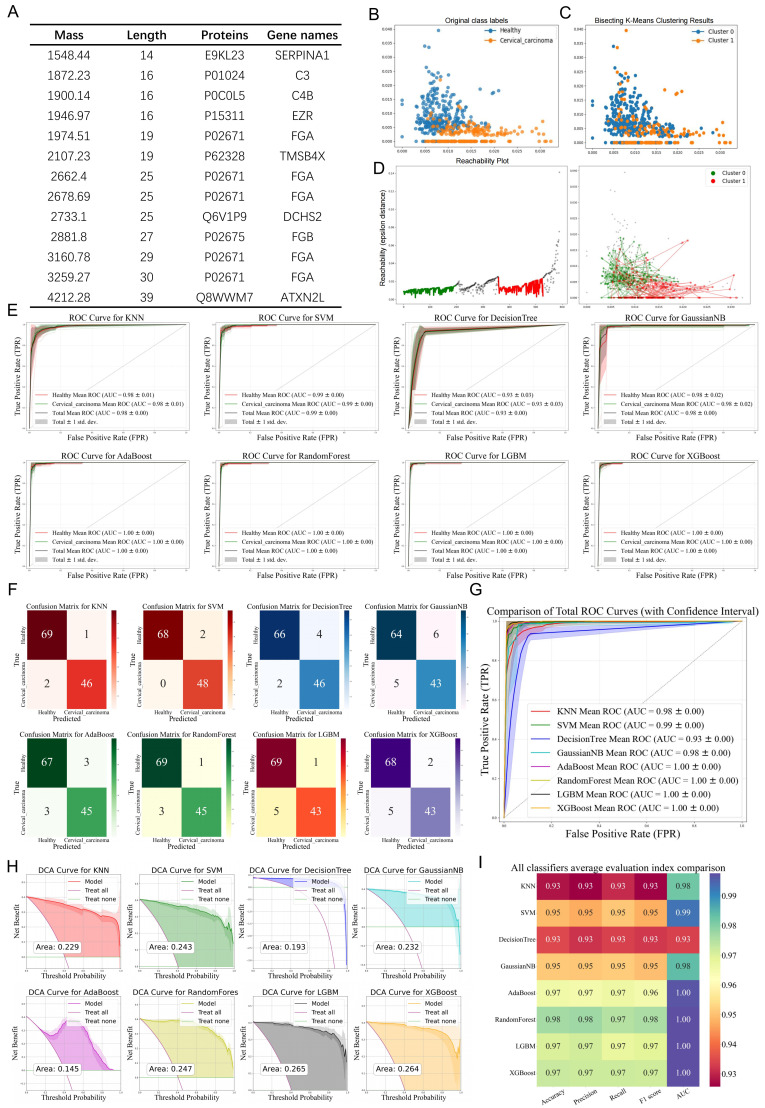
Identification of differential peptides and an evaluation of their diagnostic efficacy. (**A**) The corresponding genes of peptide peaks that differed significantly based on the UniProt database. (**B**) Original clustering, (**C**) bisecting K-means clustering, and (**D**) BIRCH clustering of the aforementioned differential genes. (**E**) ROC curves and AUC values of the differential genes under different machine learning algorithms. The shaded bands indicate the performance metric variability across cross-validation folds, highlighting model stability. (**F**) Confusion matrices under different clustering methods. The confusion matrix categorizes classification results: true positives (TP, top-left) and true negatives (TN, bottom-right) reflect correct predictions, while false positives (FP) and false negatives (FN) capture errors on the off-diagonal. (**G**) ROC curves and their association with TPR and FPR, (**H**) DCA curves, shaded areas indicate the net benefit confidence intervals across clinical thresholds. (**I**) Performance metrics including the accuracy, precision, recall, and F1 score of the differential genes under different machine learning algorithms.

Thirteen peptide peaks exhibiting significant differences between the healthy donors and cervical cancer patients were identified using LC-ESI-MS/MS and the UniProt database. The corresponding genes associated with these peptides included *SERPINA1, C3, C4B, EZR, FGA, TMSB4X, DCHS2, FGB,* and *ATXN2L* (Figure 5A). To assess the agreement between the datasets represented by these features and the original unsupervised classification, the original labels were used as a benchmark to cluster the datasets (Figure 5B). Unsupervised clustering was further performed using bisecting K-means clustering (Figure 5C) and BIRCH clustering (Figure 5D). The results demonstrated strong agreement between the unsupervised and supervised clustering, suggesting that the datasets represented by *SERPINA1, C3, C4B, EZR, FGA, TMSB4X, DCHS2, FGB,* and *ATXN2L* are suitable for further cervical cancer classification tasks (Supplementary Figure S1).

These 10 genes were selected as differential indicators, and the ROC curves along with the AUC values were generated using eight distinct machine learning methods (Figure 5E). The mean AUC values under different algorithms were as follows: Total mean AUC = 0.98 ± 0.00 (Healthy AUC = 0.98 ± 0.01, Cervical AUC = 0.98 ± 0.01), SVM: Total mean AUC = 0.99 ± 0.00 (Healthy AUC = 0.99 ± 0.00, Cervical AUC = 0.99 ± 0.00), Decision tree: Total mean AUC = 0.93 ± 0.00 (Healthy AUC = 0.93 ± 0.03, Cervical AUC = 0.93 ± 0.03), Gaussian NB: Total mean AUC = 0.98 ± 0.05 (Healthy AUC = 0.98 ± 0.02, Cervical AUC = 0.98 ± 0.02), AdaBoost: Total mean AUC = 1.00 ± 0.00 (Healthy AUC = 1.00 ± 0.00, Cervical AUC = 1.00 ± 0.00), Random forest: Total mean AUC = 1.00 ± 0.00 (Healthy AUC = 1.00 ± 0.00, Cervical AUC = 1.00 ± 0.00), LGBM: Total mean AUC = 1.00 ± 0.00 (Healthy AUC = 1.00 ± 0.00, Cervical AUC = 1.00 ± 0.00), XGBoost: Total mean AUC = 1.00 ± 0.00 (Healthy AUC = 1.00 ± 0.00, Cervical AUC = 1.00 ± 0.00).

The confusion matrix results for each classifier are presented in Figure 5F. Additionally, the ROC curves under different models were integrated to illustrate their correlation with the TPR and FPR (Figure 5G). Decision curve analysis was performed to quantify the net benefits of the prediction models under varying decision thresholds, and the corresponding DCA curves were plotted. The results indicated that the net benefits of all models exceeded the reference lines (including “treat-none” and “treat-all”), suggesting that machine learning classifiers for cervical cancer diagnosis exhibited a relatively low yield rate (Figure 5H). Key performance metrics, including accuracy, precision, recall, and F1 score, were calculated to further evaluate the models (Figure 5I).

### 3.7. Bioinformatics Analysis of Differentially Expressed Peptides

**Figure 6:**
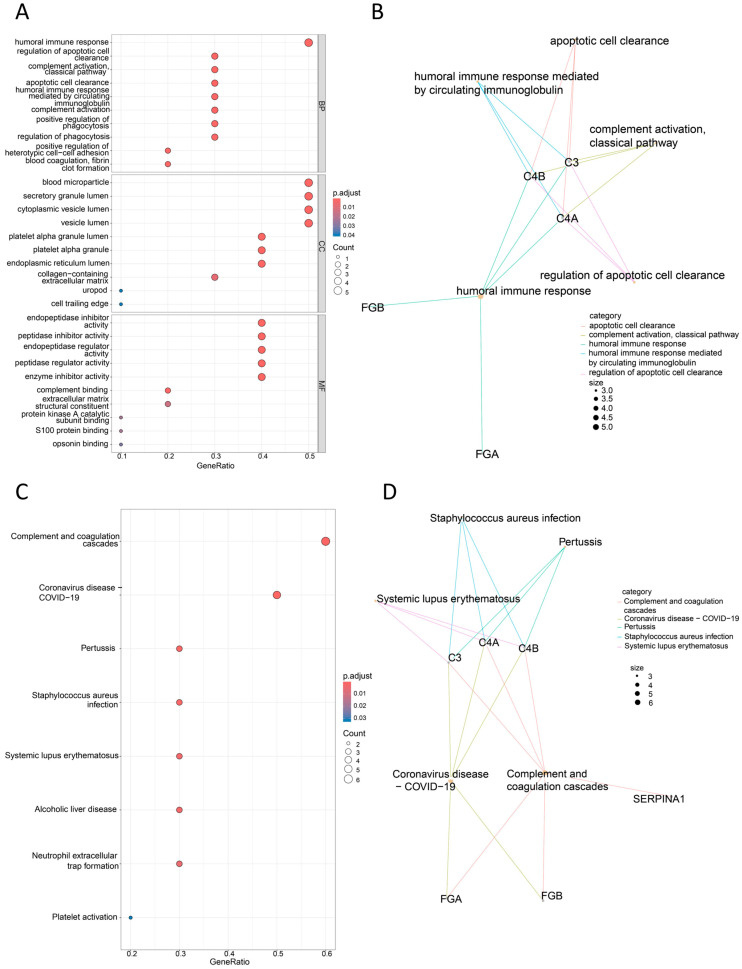
Bioinformatics analysis of differentially expressed peptides. (**A**,**B**) GO function analysis and clustering network, biological process (BP), cellular component (CC), and molecular function (MF). (**C**,**D**) KEGG pathway analysis.

**Figure 7:**
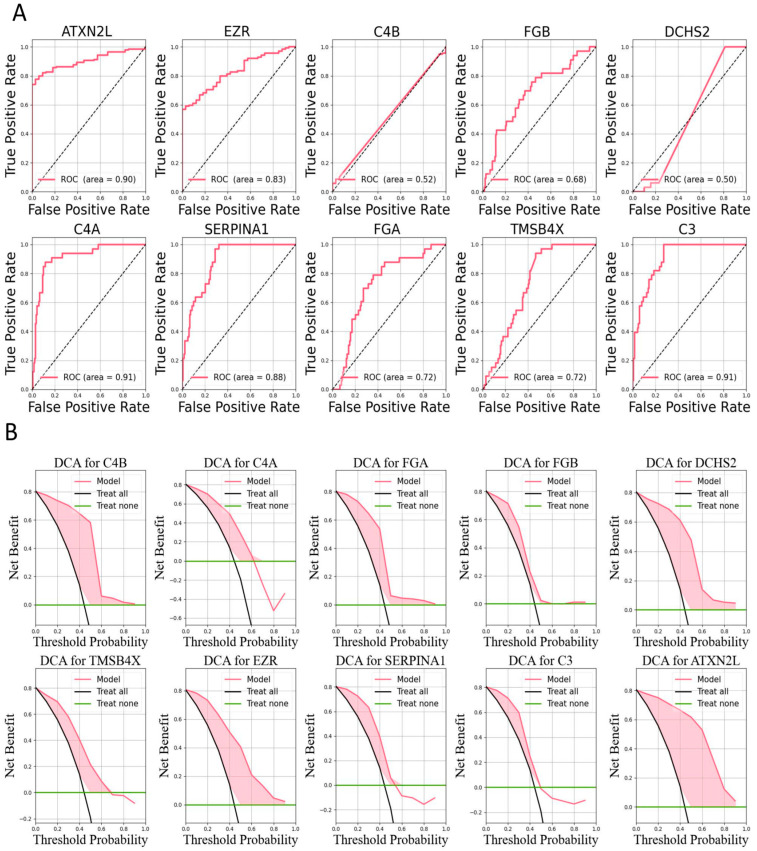
Analysis of the clinical indicators of the identified proteins. (**A**) ROC curve of single identified proteins. (**B**) DCA curve of single identified proteins and its evaluation against the reference line.

Gene Ontology (GO) and Kyoto Encyclopedia of Genes and Genomes (KEGG) functional analyses were performed on the 10 differentially identified genes using the R programming language, and clustering network graphs were generated. The GO analysis revealed that these genes are potentially involved in biological processes (BP) such as the humoral immune response, regulation of apoptotic cell clearance, complement activation, and regulation of phagocytosis. Additionally, they are associated with cellular components (CCs) including blood microparticles, secretory granule lumen, and cytoplasmic vesicle lumen as well as molecular functions (MFs) such as peptidase inhibitor activity, complement binding, and enzyme inhibitor activity (Figure 6A,B). The KEGG pathway analysis suggested that these genes may participate in disease-related pathways including complement and coagulation cascades, coronavirus disease, pertussis, *Staphylococcus aureus* infection, and alcoholic liver disease (Figure 6C,D). These analyses provide insights into the molecular activities, cellular localization, biological processes, and potential disease pathways associated with the differentially expressed peptides. Furthermore, protein expression data for these signature peptides were obtained from the PRIDE database. Cervical cancer data were extracted based on tumor classification, and ROC curves and DAC values were generated to validate the expression of individual proteins in cervical cancer (Figure 7).

## 4. Discussion

Traditional biomarker screening strategies primarily focus on identifying differences in protein abundance between experimental and control groups, typically involving three stages: biomarker discovery, confirmation, and verification, ultimately leading to the identification of clinically translatable protein biomarkers. However, candidate biomarkers derived from early proteomics studies often exhibit high false positive rates, significantly hindering subsequent clinical translation. This limitation arises because selecting individual biomarkers for ELISA validation fails to leverage the vast amount of valuable omics data generated in the early stages, increasing the likelihood of false positives and often resulting in project failure. Additionally, omics results are prone to false negatives due to factors such as high-abundance proteins (e.g., albumin, complement) masking low-abundance proteins as well as limitations in protein separation techniques, which may overlook potential disease biomarkers. To address these challenges, emerging proteomics technologies, such as targeted proteomics (e.g., PRM, Olink), have introduced strategies for identifying combinations of diagnostic markers, which have become the preferred approach. For instance, Ye et al. utilized Olink technology to analyze 1160 proteins in plasma samples from 180 Hong Kong residents aged 60 or older (106 Alzheimer’s disease (AD) patients and 74 healthy controls) and 97 Hong Kong residents aged 60 or older (36 AD patients and 61 healthy controls). They identified 19 core plasma protein markers associated with AD and developed a biomarker model capable of accurately detecting AD and assessing disease severity. Compared with existing blood biomarkers for AD classification (e.g., plasma ATN biomarkers, plasma p-tau181, and p-tau217), the combination of these 19 protein markers demonstrated superior accuracy, achieving an AUC of up to 0.98. Notably, seven of these proteins were linked to the developmental stages of AD, enabling disease staging [42]. Despite these promising results, the confirmation and validation of diagnostic markers remain challenging. Directly validating a large number of candidate markers through ELISA would entail a prohibitive workload and costs. Furthermore, the generation of high-quality antibodies based on selected diagnostic markers is a critical step in clinical translation. The specificity and affinity of these antibodies directly influence the accuracy and reliability of the detection results. Poor antibody specificity may lead to nonspecific reactions and false positives. Additionally, achieving an optimal balance between the sensitivity and specificity of ELISA kits as well as establishing stable and reliable standard curves are essential factors that impact the clinical translation and application of serum-based diagnostic markers.

Developing analytical techniques based on serum proteomics data and proposing antibody-independent diagnostic methods have emerged as promising strategies to address the aforementioned challenges. The rapid advancement of AI technology provides critical support for this approach. Specifically, by leveraging the high-throughput and high-sensitivity advantages of mass spectrometry-based proteomics, AI can be employed to identify more discriminative diagnostic markers or data features from high-dimensional proteomics data [43]. A recent study exemplifying this approach was reported by Deng et al., who analyzed the plasma proteomic data from 53,026 individuals, encompassing 2920 plasma proteins, 406 pre-existing diseases, 660 newly developed diseases during follow-up, and 986 health-related characteristics. By integrating AI and big data analytics, they identified 168,100 protein-disease associations and 554,488 protein-phenotype associations. This work comprehensively mapped the proteomic landscape of human health and disease, uncovering over 650 proteins associated with at least 50 diseases to construct disease diagnosis and prediction models. Additionally, it revealed 26 novel therapeutic drug targets [44]. In cancer research, Senuri et al. proposed a machine learning-based feature extraction workflow to identify high-performing protein markers for high-grade serous ovarian carcinoma (HGSOC) that used publicly available ovarian cancer tissue and serum proteomics datasets. They discovered several new serum proteomic biomarkers for HGSOC as a combination, demonstrating superior performance compared with known ovarian cancer biomarkers, including clinically used serum markers, with AUC values exceeding 97% in two independent cohorts [45]. In contrast to their reliance on public proteomics databases, our study began with the collection of patient sera and utilized MALDI-TOF MS to obtain differential serum polypeptide profiles between cervical cancer patients and healthy individuals. This approach offers the advantage of enabling the specific definition of study subjects, such as particular ethnicities or geographic regions, especially when differences in serum proteomics data due to genetic backgrounds are significant. Furthermore, it allows for the inclusion of multi-center patient groups at any stage of the study, expanding the serum proteomics database and yielding a more accurate and reliable analytical model. Another distinction lies in our methodology: we employed eight distinct machine learning models to jointly perform the classification analysis of differential polypeptide peaks. ROC curves and confusion matrices were constructed, and key performance metrics, including accuracy, precision, recall, and F1 score, were calculated. The results revealed a low yield rate for the clinical diagnosis of cervical cancer using machine learning classifiers. Based on these findings, we focused on classifiers with the feature_importances_ attribute such as decision tree, random forest, light Gradient boosting machine, and extreme gradient boosting. Feature importance values were extracted and cross-validated, identifying three significant differential polypeptide peaks: M/Z = 2881.80, M/Z = 1900.14, and M/Z = 5355.20. Additionally, Shapley values and LIME values were computed under different models. By integrating these three importance indicators—feature importance values, Shapley values, and LIME values—we optimized the learning model from multiple dimensions.

To evaluate the reliability of the multi-dimensional learning model, potential high-performing polypeptides were identified using LC-ESI-MS/MS and the UniProt database, revealing genes such as *SERPINA1, C3, C4B, EZR, FGA, TMSB4X, DCHS2, FGB*, and *ATXN2L*. PRIDE protein expression analysis, along with KEGG and GO functional analyses, were conducted on these genes. For instance, the *DCHS2* (Dachsous cadherin-related 2) gene, located on human chromosome 4q31.3, encodes a protein containing multiple cadherin domains that may play a role in intercellular adhesion and signaling. In colorectal cancer, *DCHS2* expression is significantly elevated compared with adjacent normal tissues, and its abnormal expression is strongly correlated with prolonged progression-free survival [46]. These findings validate the reliability of the potential polypeptides selected by our machine learning model from a functional genomics perspective.

Nevertheless, several limitations of this study should be acknowledged. First, additional multi-center proteomic datasets, including samples from cervical cancer patients and healthy donors, are needed to validate the generalizability of our model, particularly its sensitivity, specificity, and accuracy. Our current dataset primarily reflects the genetic and demographic characteristics of the local population in China, which may introduce geographic and ethnic homogeneity biases. This regional specificity could limit the generalizability of the model to populations with distinct genetic profiles, environmental exposures, or healthcare practices. Future work will prioritize collaborations with international institutions to incorporate multi-ethnic datasets, thereby enhancing the model’s generalizability and reducing bias. Second, the KEGG and GO functional analyses indicated that the differentially expressed peptides identified by our model are involved in diverse molecular processes and disease pathways. Further studies are required to determine whether these biological processes are directly linked to the pathogenesis of cervical cancer. Third, while our study highlights the potential of ML in cervical cancer diagnosis, its successful translation into clinical practice requires a multi-faceted approach. This may include rigorous validation, seamless integration into clinical workflows, and adherence to evolving regulatory and ethical standards. Finally, we propose that integrating multi-omics data (e.g., transcriptomics and metabolomics) may provide more accurate biomarker screening for disease diagnosis compared with single-omics approaches. Building on this study, future research should explore whether our learning model can be adapted to incorporate integrated analyses of other omics data, which remains a promising direction for further investigation.

## 5. Conclusions

The application of machine learning in cervical cancer diagnostics has garnered significant attention in recent years, with numerous studies exploring the potential of ML algorithms to improve early detection, risk stratification, and treatment planning. Previous research has primarily focused on leveraging various data modalities, including imaging, genomic, and proteomic data, to develop predictive models. For instance, studies utilizing Pap smear images have demonstrated the feasibility of automated image analysis for detecting precancerous lesions, achieving promising accuracy rates. Similarly, genomic studies have identified molecular signatures associated with cervical cancer progression, offering insights into disease mechanisms and potential therapeutic targets.

However, several limitations persist in the existing literature. First, many studies rely on small, homogeneous datasets, which restrict the generalizability of their findings. The lack of diversity in patient populations, particularly in terms of age, ethnicity, and geographic location, raises concerns about the applicability of these models to broader clinical settings. Second, the integration of multi-omics data (e.g., genomic, proteomic, and transcriptomic) remains underexplored, despite its potential to provide a more comprehensive understanding of cervical cancer biology. Third, while ML models have shown high accuracy in controlled environments, their performance often degrades when applied to real-world clinical data due to variations in data quality, acquisition protocols, and preprocessing methods. Fourth, the interpretability of ML models remains a significant challenge, as many algorithms operate as “black boxes”, limiting their adoption by clinicians who require transparent and explainable decision-making tools.

In conclusion, our study established an effective mass spectrometry-based proteomics data workflow for the discovery and validation of serum biomarkers for the early diagnosis of cervical cancer by leveraging machine learning capabilities. We identified a panel of high-performance novel biomarkers with significant diagnostic potential and functional relevance, which may serve as a valuable reference for the early diagnosis and development of novel therapeutic interventions for cervical cancer.

## Data Availability

The datasets generated and/or analyzed during this study are available from the corresponding author upon reasonable request.

## Supplementary Materials

The following supporting information can be downloaded at: https://www.mdpi.com/article/10.3390/bioengineering12030269/s1, Figure S1: Their Diagnostic Efficacy of Selected Differential Peptides. (A) 2D/3D visualization of selected differential peptides after dimensionality reduction using PCA, KPCA, and t-SNE. (B) Shapley and (C) LIME analysis for selected differential peptides.
