# Supplementary material for: Integrated Machine Learning Algorithms-Enhanced Predication for Cervical Cancer from Mass Spectrometry-Based Proteomics Data
Source: Bioengineering (Basel). 2025 Mar 9;12(3):269. doi: 10.3390/bioengineering12030269 (PMC11939187; doi:10.3390/bioengineering12030269)
Supplement: Supplementary file 1 [file bioengineering-12-00269-s001.zip › bioengineering-3464576-supplementary.pdf]

bioengineering

MDPI

Supplementary Figure S1

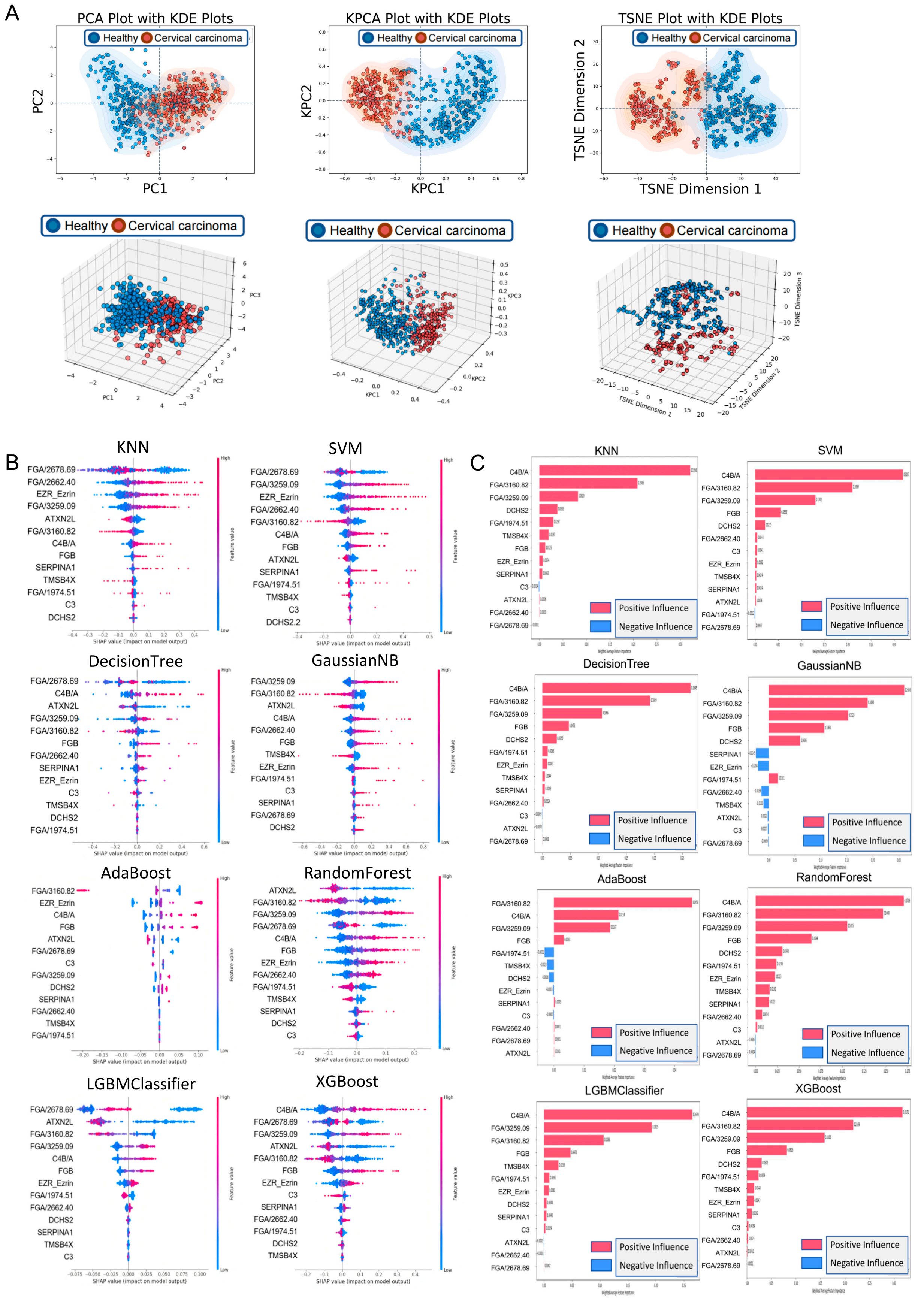

Figure S1. Their Diagnostic Efficacy of Selected Differential Peptides. (A) 2D/3D visualization of selected differential peptides after dimensionality reduction using PCA, KPCA, and t-SNE. (B) Shapley and (C) LIME analysis for selected differential peptides.
